# Structural Interventions in Heart Failure: Mending a Broken Heart

**DOI:** 10.3390/jcm12093243

**Published:** 2023-05-01

**Authors:** David Katzianer, Chonyang Albert

**Affiliations:** Department of Cardiovascular Medicine, Cleveland Clinic Foundation, Cleveland, OH 44195, USA

**Keywords:** heart failure, cardiomyopathy, structural heart disease, valvular heart disease, percutaneous, intervention

## Abstract

Advanced heart failure is often accompanied by perturbations in cardiac chamber or valve geometries which result in worsening cardiac function and hemodynamics. Once limited to surgical procedures, recent developments in minimally invasive percutaneous techniques have demonstrated efficacy in patients with both reduced and preserved ejection fraction who are at an elevated surgical risk for perioperative events. This review highlights a subset of the interventions available in clinical practice or in development for the treatment of these valvular and structural alterations.

## 1. Introduction

Despite advancements in medical and procedural therapies, the overall prevalence of heart failure (HF) is increasing. Statistics from the American Heart Association in 2021 estimated that the prevalence of heart failure in the United States is approximately 6 million patients ≥20 years old, with the total percentage of heart failure patients projected to increase from 2.4% of the US population in 2012 to 3.0% in 2030 [1]. Corresponding to this rise in prevalence is an increase in the overall costs of HF on the U.S. economy, with an estimated USD 30.7 billion of healthcare dollars spent in 2012 and a projected increase to USD 69.8 billion in 2030 [1]. These increases are coupled with a shift in the landscape of heart failure patient characteristics: a review of the data from the Framingham Heart Study (FHS) and Offspring Cohorts and the Cardiovascular Health Study (CHS) observed an increased incidence of preserved ejection fraction from 2000 to 2009 when compared to 1990–1999 (incidence rate ratio 1.53, *p* < 0.0001), along with a decreased incidence ratio for HFrEF of 0.80 during that same period (*p* = 0.0029) [2].

**Figure 1:**
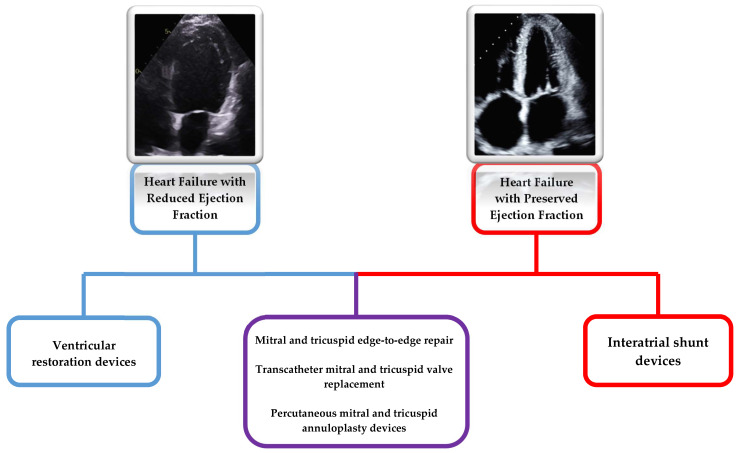
Structural interventions in heart failure with reduced and preserved ejection fraction.

Congestive heart failure is often accompanied by structural alterations in cardiac chamber dimensions or valvular geometry, and these perturbations have been the subject of numerous therapeutic interventions in various phases of clinical investigation. Treatment of patients with structural heart disease necessitates the implementation of heart failure guideline-directed medical therapies (GDMT) along with optimal intravascular volume optimization. The ACC/AHA/HFSA published an updated 2022 guideline summarizing recommendations for the optimization of HF GDMT, with recommendations based on ejection fraction [3]. Despite optimized medications and heart failure status, a subset of patients with structural heart disease will remain moderately to severely symptomatic. Historically, treatment has been limited to medical optimization and surgical techniques; however, the advancements of hybrid and fully percutaneous therapies have enabled the increased utilization of structural interventions as a targeted approach for heart failure patients often regardless of the ejection fraction with some of these therapies now referenced in the 2020 ACC/AHA guidelines for management of valvular heart disease [4]. In this article, we review a subset of common structural interventions for patients with heart failure, highlight the available evidence for these procedures, and offer a glimpse into future interventions that are currently under investigation. A summary of these devices across the spectrum of heart failure can be found in Figure 1 and Table 1.

## 2. Valvular Heart Disease

### 2.1. Mitral Regurgitation

Secondary or functional mitral regurgitation (FMR) is common in heart failure due to poor leaflet coaptation resulting from left ventricular dysfunction, annular dilatation, left atrial enlargement, or tethering of the mitral valve leaflets [25]. Recent observational data have indicated that the incidence of severe secondary mitral regurgitation increases with the reduction in ejection fraction (EF) and is present in approximately 4.5% of those with preserved EF compared to 25% of those with reduced EF [26]. Mitral regurgitation in and of itself results in deleterious hemodynamic effects and contributes to adverse LV remodeling [27]. The severity of FMR has been linked to an increased 5-year mortality among heart failure patients, with moderate FMR carrying an unadjusted hazard ratio of 1.29 (95% CI 1.20 to 1.38, *p* < 0.001) and severe FMR of 1.82 (1.64 to 2.02, *p* < 0.001) when compared to HF patients with no or mild MR [26]. Retrospective studies have shown that patients with severe FMR also have significant symptomatic limitations (OR = 1.8, 95% CI 1.1 to 2.8, *p* = 0.01) and hospitalization for decompensated heart failure (HR = 1.7, 95% CI 1.2 to 2.5, *p* = 0.004) compared to patients without FMR [28]. Surgical data suggest improvements in functional status, LV end-systolic volume index, mitral regurgitant volume, and BNP at one year in patients with FMR undergoing CABG with mitral valve replacement when compared to those undergoing CABG alone [29]. Recent AHA/ACC guidelines list a class-IIa recommendation for mitral valve surgery in symptomatic patients with severe FMR undergoing CABG [4]. Recently, transcatheter mitral valve interventions have provided additional options for patients deemed high-risk for conventional surgery, traditionally defined as a ≥8% 30-day surgical mortality on the Society of Thoracic Surgeons (STS) risk score. Percutaneous mitral valve techniques can be broadly categorized into edge-to-edge leaflet repair, annuloplasty, and mitral valve replacement.

While the most robust data exist for functional mitral regurgitation, percutaneous mitral valve therapies may be considered in patients with primary mitral regurgitation with favorable anatomy or in patients with degenerated surgical prostheses. The 2020 ACC/AHA *Guideline for the Management of Patients with Valvular Heart Disease* lists a class-IIa recommendation for transcatheter edge-to-edge repair in patients with severe primary MR with NYHA class III/IV symptoms and a prohibitive surgical risk [4]. The “Percutaneous MitraClip Device or Surgical Mitral Valve REpair in PAtients with PrImaRy MItral Regurgitation Who Are Candidates for Surgery” (REPAIR MR) trial is currently in process to compare percutaneous transcatheter edge-to-edge repair with surgical repair in patients with severe primary mitral regurgitation who are at a moderate surgical risk [30].

### 2.2. Edge-to-Edge Mitral Valve Repair

The 2020 AHA/ACC *Guideline for the Management of Patients with Valvular Heart Disease* and the 2022 AHA/ACC/HFSA *Guideline for the Management of Heart Failure* list a class-IIa recommendation for transcatheter edge-to-edge mitral valve repair in patients with systolic dysfunction and severe symptomatic functional mitral regurgitation despite optimal guideline-directed medical therapy [3,4]. Although these recommendations specifically address patients with reduced systolic function, recent studies have shown an improvement in MR grade, NYHA functional class, and heart failure hospitalizations among HFpEF patients who underwent percutaneous edge-to-edge repair for moderate-to-severe mitral regurgitation [6,31].

### 2.3. MitraClip

At the time of this publication, the MitraClip device (Abbott Medical Devices, Santa Rose, CA, USA) is the most prevalent transcatheter mitral valve repair technique used in the United States [32]. This technique is based on the surgical Alfieri stitch method of anchoring the anterior and posterior mitral valve leaflet edges to each other, resulting in a double-orifice mitral valve to minimize the regurgitant volume, also known as an edge-to-edge repair [32,33]. The largest clinical trial to date (COAPT) enrolled symptomatic patients with moderate-to-severe or severe FMR and showed a lower rate of heart failure hospitalization at 24 months (HR 0.53, 95% CI 0.40 to 0.70; *p* < 0.001) and all-cause mortality at 24 months (HR 0.62, 95% CI 0.46 to 0.82, *p* < 0.001) in patients undergoing MitraClip compared to optimal medical therapy alone [5]. The MitraClip received FDA approval for the treatment of degenerative MR in 2013 and for the treatment of secondary MR in 2019.

### 2.4. PASCAL

The PASCAL precision transcatheter valve repair system (Edwards Lifesciences LLC, Irvine, CA, USA) was approved by the FDA in September 2022 as another edge-to-edge repair option for patients with significant mitral valve regurgitation. In the CLASP and CLASPIID trials, this device demonstrated a sustained reduction in mitral valve regurgitation at 2 years and similar rates of device implant complications when compared to the MitraClip [7,34].

### 2.5. Transcatheter Mitral Annuloplasty

There are several investigational devices seeking to reduce mitral regurgitation through annuloplasty-like effects. These include the Carillon mitral-contour system (Cardiac Dimension Inc., Kirkland, WA, USA), Cardioband (Edwards Lifesciences, Irvine, CA, USA), Accucinch (Ancora Heart, Santa Clara, CA, USA) and Mitralign (Mitralign, Tewksbury, MA, USA). These devices are not approved for commercial use in the United States [35].

### 2.6. Carillon Device

The Carillon mitral-contour system (Cardiac Dimension Inc., Kirkland, WA, USA) is a percutaneous mitral annuloplasty device that is placed in the coronary sinus to reduce mitral annular diameter by utilizing the close proximity of the posterior annulus and coronary sinus [10]. Trials investigating the use of the Carillon device have been small but have shown favorable reductions in regurgitant volume and left ventricular dimensions; outcomes on functional data such as the 6-min walk test and Kansas City (KCCQ) scores have been conflicting [8,9,10]. A pooled analysis of these three trials showed a significant improvement in the echocardiographic parameters of LV and mitral valve morphology, reduction in MR grading, improvement in Kansas City Cardiomyopathy Questionnaire score, and heart failure hospitalization in the Carillon device group [36]. Leaflet morphology is unchanged by the Carillon percutaneous mitral annuloplasty device, and thus subsequent mitral valve interventions are theoretically possible [32].

### 2.7. Cardioband Device

The Cardioband device (Edwards Lifesciences, Irvine, CA, USA) is a trans-septal mitral annuloplasty system consisting of a band which is anchored along the posterior annulus and contracted to reduce the septal–lateral dimension of the annulus, thereby reducing mitral regurgitation [32]. A single-arm prospective trial has shown durable reductions in mitral regurgitation severity and significant improvement in functional status at one year [11]. The ACTIVE trial (NCT03016975) is currently ongoing to compare the Cardioband device in conjunction with medical therapy to optimal medical therapy alone.

### 2.8. Mitralign

The Mitralign system utilizes pledgeted sutures anterior and posterior to the mitral valve commissures to reduce annular dimensions via cinching the sutures [32]. The Mitralign percutaneous annuloplasty first-in-man study demonstrated significant improvements in left ventricular volumes and mitral valve geometry and improvements of the 6-min walk distance and NYHA functional class at 6 months [37].

### 2.9. Transcatheter Mitral Valve Replacement (TMVR)

Surgical literature has demonstrated a more durable response to mitral valve replacement compared to mitral valve repair in patients undergoing surgical intervention for ischemic mitral regurgitation [38,39]. Unfortunately, many heart failure patients are at increased or prohibitive surgical risk, and thus transcatheter options for valve replacement are under investigation. The development of a percutaneous mitral valve replacement system is complicated by numerous technical challenges, including the position and geometric constraints of the saddle-shaped native mitral valve, a complex subvalvular apparatus, concern for the development of left ventricular outflow tract obstruction, and paravalvular leaks, and anchoring the replacement valve to the dynamic mitral annulus [32,35,40]. Current literature supports the use of transcatheter valve-in-valve procedures in the setting of degenerated bioprosthetic valves, with higher mortality and complications associated with patients undergoing transcatheter mitral valve-in-ring or valve-in-MAC interventions [40].

There are numerous additional TMVR systems currently under investigation for transseptal or transapical native mitral valve replacement [35,40]. The largest data exist for the transapical Tendyne mitral-valve system (Abbott Vascular, Roseville, MN, USA) with a recent retrospective review of 108 high-surgical-risk patients with moderate-to-severe MR demonstrating encouraging technical and procedural results along with a near-to-complete resolution of MR and improvement of the NYHA class among patients [12,13]. Fifty six percent of the patients who underwent valve implantation had an LVEF ≥50% (mean LVEF 48% ± 12), though these outcomes data are not stratified for patients with preserved systolic function [13]. The Sapien 3 transcatheter heart valve (Edwards Lifesciences) has shown promising and durable improvements in functional symptoms in patients with moderate-to-severe MR or severe mitral stenosis at 1-year post-implantation [14,15]. In 2021, the Tendyne mitral valve system received FDA breakthrough-device designation in patients with severe mitral annular calcification, and the Sapien 3 valve system was FDA-approved for valve-in-valve replacement of bioprosthetic mitral valves or a valve-in-ring native mitral valve with an annuloplasty ring. A detailed recent review of the available TMVR systems including procedural success, mortality rates, and mitigation strategies for LVOT obstructions can be found from Nagaraja et al. [40].

### 2.10. Tricuspid Valve

Secondary or functional tricuspid regurgitation (TR) is the most common etiology for tricuspid valve disease in Western countries and is frequently associated with left-sided heart failure or pulmonary hypertension leading to RV dilation and dysfunction with resultant tricuspid annular dilation [41]. The poor prognosis of TR has long been noted, with studies showing a relative mortality risk of 1.55 (95% CI 1.14–2.11) for patients with LVEF ≤35% and severe tricuspid regurgitation with increasing 1-year and 3-year mortality associated with increasing TR severity [42]. Despite this, TR has traditionally been conservatively managed with a focus on treating the underlying heart failure. A retrospective review of >500 patients with severe TR and heart failure from our institution has reported a decreased mortality associated with patients who underwent tricuspid valve surgery compared with those managed medically (HR 0.44, 95% CI 0.27 to 0.71) [43]. The 2020 ACC/AHA *Guideline for the Management of Patients with Valvular Heart Disease* lists a class-I recommendation for tricuspid valve surgery in patients with severe TR undergoing left-sided surgery and a class-IIa recommendation for surgery in patients with isolated severe TR and right-sided heart failure [4]. Recent improvements in transcatheter technologies have shifted the paradigm for tricuspid valve interventions due to the exceedingly high- or prohibitive surgical risk of these patients [41]. A detailed recent review of investigation transcatheter therapies can be found from Alperi et al. [44]. Transcatheter strategies to address the tricuspid valve include annuloplasty devices, edge-to-edge repair, valve replacement, and caval systems.

### 2.11. Tricuspid Annuloplasty Devices

Several devices have been investigated for reducing the tricuspid annular diameter. These include suture annuloplasty devices such as the TriAlign and TriCinch systems, as well as direct ring annuloplasty devices such as the Cardioband and Millipede devices. The TriAlign system (Mitralign Inc., Tewksbury, MA, USA) is a replication of the modified Kay surgical bicuspidization procedure; an early feasibility study of the TriAlign system demonstrated improvement in the echocardiographic parameters of tricuspid annular area and EROA, as well as in patients’ functional status at 30 days [17]. The TriCinch system (4Tech Cardio Ltd., Galway, Ireland) is a percutaneous annuloplasty device that reduces the septolateral dimensions to remodel the anteroposterior annulus; there have been reports of successful implantation with a reduction in tricuspid regurgitation; however, the results of an 18-patient prospective trial (NCT03294200) are currently pending [45,46].

The Cardioband system (Edwards Life Sciences, Irvine, CA, USA) is a transcatheter tricuspid implant that is designed to reduce tricuspid regurgitation via a reduction in the annular dimension. The prospective TRI-REPAIR (Tricuspid Regurgitation Repair with Cardioband Transcatheter System) was a single-arm trial that investigated the use of the Cardioband system for treatment of severe tricuspid regurgitation in 30 patients (LVEF mean 57.2 ± 10.5%; range 29.5 to 71.0%) [47]. There were significant reductions in echocardiographic assessment of tricuspid regurgitation at 30-days, along with improvement in functional improvements in NYHA classification (*p* < 0.0001), 6-min walk distance (266 to 326 m, *p* = 0.0035), and KCCQ score (46 to 70, *p* < 0.0001) at 6 months [47]. One year results from The Edwards Cardioband tricuspid valve reconstruction system early feasibility study has shown durable improvements in tricuspid regurgitation (73.1% of patients with ≥2 grade TR reductions) and functional status (92.3% of patients achieving NYHA class I/II symptoms, *p* < 0.0001) among patients who underwent the procedure [16].

### 2.12. Edge-to-Edge Tricuspid Valve Repair

The TriClip device (Abbott, Chicago, IL, USA) is a transcatheter edge-to-edge approximation device which is deployed via a transfemoral delivery system for the percutaneous treatment of tricuspid regurgitation [48]. The initial data from the TriValve registry included 249 patients with severe TR treated with edge-to-edge repair and showed improvements in TR severity (TR <2+ in 72% of patients) and functional status (NYHA class <II in 69% of patients) at one year [19]. The original TRILUMINATE trial was a single-arm, prospective trial that investigated the use of the TriClip tricuspid valve repair system in 85 patients with moderate or severe TR. One-year results showed a significant reduction in TR to moderate or less in 71% of patients (*p* < 0.0001) along with functional improvements in NYHA class (83% vs. 31%, *p* < 0.0001), 6-min walk distance (303.2 vs. 272.3 m, *p* = 0.0023), and KCCQ score (improvement of 20, *p* < 0.0001) when compared to baseline results [18]. The TRILUMINATE pivotal trial is a multi-center, randomized trial of optimal medical therapy versus transcatheter edge-to-edge repair (TEER) with TriClip for treatment of severe tricuspid valve regurgitation [49]. The primary endpoint of this trial was a hierarchical composite endpoint including all-cause death or tricuspid valve surgery, heart failure hospitalization, and improvement in the Kansas City (KCCQ) with an improvement of at least 15 points in the KCCQ for heart failure-related quality of life. The results favored TEER with a win ratio of 1.48 (95% confidence interval 1.06–2.13, *p* = 0.02); however, there was no statistically significant difference in death or tricuspid valve surgery nor in the rate of heart failure hospitalizations. The KCCQ score changed by a mean of 12.3 ± 1.8 points in the TEER group compared to 0.6 ± 1.8 in the control group (*p* < 0.001). There was also a significant reduction in tricuspid regurgitation at 30 days post procedurally. These data support the efficacy and safety of TR reduction and indicate an improvement in heart-failure quality of life with reduction in TR.

Although the TriClip device is the most thoroughly studied tricuspid edge-to-edge repair device, recent study results for the PASCAL repair system (Edwards Lifesciences, Irvine, CA, USA) in 235 high-risk patients demonstrated successful TR reduction to moderate or less in 78% of patients (*p* < 0.001) with an improvement of right ventricular end-diastolic diameter (56 vs. 53 mm, *p* < 0.001) and NYHA functional class at follow-up (*p* < 0.001) [50].

### 2.13. Transcatheter Tricuspid Valve Replacement (TTVR)

A number of tricuspid valve-replacement systems are currently under investigation as minimally-invasive options via either transvenous or transatrial approaches through mini-thoracotomy [48]. Initial data suggest that these therapies are associated with significant reductions in tricuspid regurgitation and improvement in functional status, though large-scale investigations and long-term outcomes are currently lacking [48,51]. Detailed recent reviews of available transcatheter tricuspid valve-replacement systems and 30-day patient outcomes for respective devices can be found from Romeo et al. and Alperi et al. [44,48].

Additional strategies for the mitigation of tricuspid regurgitation have been explored using percutaneous implantation of prosthetic valves into either the inferior vena cava or bicaval positioning to functionally reduce regurgitation into the systemic venous system. Currently employed valves include the balloon-expandable Sapien XT or Sapien 3 valves (Edwards Lifesciences, USA) following pre-stenting of the caval veins or the self-expanding TricValve system (Products + Features, Germany). Neither of these devices are currently approved for this indication; however, they have been performed under compassionate use or in clinical studies [52]. The TricValve bicaval technique has shown promising improvements in quality of life and NYHA functional class at 6 months [20].

## 3. Ventricular Restorative Devices

Heart failure with reduced ejection fraction results in progressive deleterious left ventricular remodeling leading to increased end-diastolic volumes, myocardial stress, functional mitral regurgitation, and worsening heart failure symptoms [53]. Early surgical techniques of left ventricular geometry restoration have been met with mixed results; however, this adverse remodeling of the left ventricular cavity has recently generated renewed interest as a target of percutaneous left ventricular restoration devices [54].

### 3.1. Parachute Device

The Parachute device (CardioKinetix, Redwood City, CA, USA) is a conical nitinol frame covered with a fluoropolymer membrane that can be delivered into the left ventricular cavity via a percutaneous approach to partition off an akinetic or dyskinetic left ventricular apex [21]. Initial trials of its use in 39 patients with ischemic cardiomyopathy, NYHA class II to IV symptoms, an LVEF between 15 to 40%, and dilated akinetic or dyskinetic anterior–apical walls showed an improvement or maintenance of NYHA functional class in 85% of patients, as well as improvement in LVEDV*i* (*p* = 0.0056) at 3-year follow up [21]. Results have been mixed across the PARACHUTE trials and more research is necessary to determine the safety and outcomes associated with these devices; a detailed review of the Parachute device can be found from Dhakal and Oliveira [55]. This Parachute device is not currently in use [53].

### 3.2. AccuCinch

Another device currently under investigation for the reverse remodeling of altered left ventricular geometry is the AccuCinch (Ancora Heart, Inc) ventricular restoration system. This device is a nitinol anchor-based cinching cable that is percutaneously placed in the left ventricular cavity below the level of the mitral valve, functioning to reduce the size of the left ventricle [53]. Early feasibility data suggest an improvement in symptomatology and LVEDV at 12-months post-device implantation according to the manufacturer [56]. The CORCINCH-HF study (ClinicalTrials.gov identifier: NCT04331769) is a prospective, randomized, open-label study that is currently enrolling patients with an LVEF 20–40%, LVEDD ≥ 55 mm, and NYHA II–IV symptomatology to undergo optimal medical therapy compared to device + OMT.

### 3.3. Revivent TC System

The Revivent TC system (BioVentrix, Mansfield, MA, USA) is a left ventricular restoration device which functions to exclude scarred myocardium in the anteroseptal, anterolateral, or apical regions; this device is comprised of an internal anchor placed in the right ventricular septum via internal jugular venous access and external anchors placed in the LV epicardial wall via a left lateral thoracotomy approach [22]. These anchors are then drawn together via a poly-ether-ether-ketone tether to exclude a portion of scarred myocardium [22]. An initial prospective, single-arm study investigating the use of the Revivent TC system in 89 patients with NYHA II–IV symptoms and LV dilatation or dysfunction due to scarring showed a significant improvement in LVEF (29 vs. 34%, *p* < 0.005) and a reduction in LVESV*i* (74 vs. 24 mL/m^2^, *p* < 0.0001) and LVEDV*i* (106 vs. 80 mL/m^2^, *p* < 0.0001) at one year, along with improvements in NYHA functional class, 6-min walk distance, and the Minnesota Living with Heart Failure Questionnaire scores [22].

## 4. Interatrial Shunting Devices

Dyspnea in heart failure patients is largely related to pulmonary venous congestion as a result of elevated left atrial pressures. Prior studies have reported an increased 10-year mortality among patients with suspected HFpEF and an elevated resting pulmonary capillary wedge pressure (PCWP) and for those with a low resting PCWP but increased exercise PCWP [57]. A more recent retrospective study of patients with both HFrEF and HFpEF have similarly found an association of increased mortality with elevated PCWP at peak workload relative to body weight [58]. With this, several devices have been developed with the purpose of decompressing the left atrium via the creation of a left-to-right atrial shunt.

The Interatrial Shunt Device (IASD, Corvia Medical, Tewksbury, MA, USA) is a self-expanding nitinol cage that is placed percutaneously via the femoral vein to create an 8-mm-diameter communication between the atria [59]. The initial data in the REDUCE-LAP I trial 1-year follow-up data demonstrated the safety and patency of the device at 1-year, as well as promising results in hospitalization (22% in IASD versus 63% in control, *p* = 0.06) [24]. The largest trial to date was the REDUCE-LAP II trial which randomized 626 patients with LVEF >/= 40% and exercise-induced PCWP elevation to either the atrial shunt device or a sham procedure in a double-blinded fashion and did not show significant benefit in the primary composite endpoint of cardiovascular death or non-fatal ischemic stroke at 12 months, rate of total heart failure events up to 24 months, and change in KCCQ score at 12 months [23]. The V-wave shunt device (V-wave Ltd., Caesarea, Israel) is a percutaneously delivered interatrial shunt system currently under investigation in a prospective, randomized study as part of the “Reducing Lung Congestion Symptoms in Advanced Heart Failure” (RELIEVE-HF) trial (NCT03499236). Additional shunt devices are being investigated. A more comprehensive review of interatrial shunt devices in the treatment of advanced heart failure can be found from Emani et al. and Yum and Frishman [60,61].

## 5. Conclusions

Structural perturbations represent both a cause and consequence of the deleterious hemodynamic effects of congestive heart failure. Valvular disease and adverse ventricular remodeling occur across the spectrum of left and right ventricular ejection fraction, and heart failure patients are frequently among the highest adverse surgical risk profiles. In this review, we highlight the advances in minimally invasive approaches to valvular heart disease, ventricular-geometry restoration, and intra-cardiac shunting for patients with heart failure with reduced and preserved ejection fractions.

## Figures and Tables

**Table 1:** Structural interventions in heart failure.

	Device	Implantation Approach	Population	Results	References
Mitral Valve					
Edge-to-Edge Repair					
	MitraClip (Abbott Medical, USA)	Femoral vein	HFrEF (LVEF 20–50%) Moderate-to-severe or severe functional MR	↓ HF hospitalization at 24 months (annualized rate, HR 0.53, *p* < 0.001) ↓ MR severity in device group Improvement in NYHA functional class, KCCQ score at 12 months Improvement in LVEDV at 12 months ↓ All-cause mortality at 24 months (HR 0.62, *p* < 0.001)	[5]
	MitraClip (Abbott Medical, USA)	Femoral vein	HFpEF (LVEF > 50%) Degenerative and functional MR	↓ MR severity at 12 months Improvement in NYHA functional class at 12 months ↓ HF hospitalization at 12 months	[6]
	PASCAL (Edwards Lifesciences LLC, USA)	Femoral vein	LVEF ≥ 20% MR grade ≥ 3+ NYHA class II–III Degenerative and functional MR	↓ MR severity at 24 months in both DMR and FMR ↓ LVEDV at 24 months in both DMR and FMR Improvement in NYHA functional class at 24 months in both DMR and FMR ↓ Annualized rate of HF hospitalization in both DMR and FMR	[7]
Annuloplasty Devices					
	Carillon device (Cardiac Dimension Inc, USA)	Right internal jugular vein	LVEF < 40%, functional MR (TITAN I) LVEF < 40%, functional MR (TITAN II) LVEF < 50%, functional MR (REDUCE FMR)	↓ Regurgitant volume (TITAN I, TITAN II, REDUCE FMR) ↓ MR Echocardiographic quantitative parameters (TITAN I, TITAN II) ↓ LVEDV (TITAN I, REDUCE FMR) ↓ LVESV (TITAN I, REDUCE FMR) ↑ 6MWD (TITAN I) ↑ KCCQ (TITAN I) ↓ Mitral annular diameter (TITAN II)	[8,9,10]
	Cardioband device (Edwards Lifesciences, USA)	Femoral vein	LVEF ≥ 25% Moderate or severe functional MR	↓ MR severity at 12 months Improvement in NYHA functional class at 12 months ↑ 6MWD at 12 months	[11]
Transcatheter Mitral-Valve Replacement (TMVR)					
	Tendyne mitral-valve replacement system (Abbott, USA)	Transapical	Symptomatic moderate-to-severe MR	↓ MR severity at 24 months ↓ RV systolic pressure at 24 months ↓ NYHA functional class ↓ Annualized rate of HF hospitalization	[12,13]
	Sapien 3 mitral-valve replacement system (Edwards Lifesciences, USA)	Femoral vein	Valve-in-MAC, valve-in-ring, valve-in-valve LVEF ≥20% NYHA functional class II or greater symptoms Severe mitral stenosis Moderate-to-severe MR	↓ NYHA functional class at 12 months	[14,15]
Tricuspid Valve					
Annuloplasty Devices					
	TriAlign (Mitralign Inc, USA) TriCinch (4Tech Cardio Ltd., Ireland) Cardioband (Edwards Lifesciences, USA) Millipede device (Boston Scientific, USA)	Internal jugular vein (TriAlign) Femoral vein (Cardioband) Femoral vein (TriCinch)	NYHA functional class ≥ II and moderate or greater functional TR (TriAlign) ≥Moderate TR (Cardioband)	↓ Tricuspid dimensions at 30 days (TriAlign) and 12 months (Cardioband) ↓ EROA at 30 days (TriAlign) and 12 months (Cardioband) ↑ LV stroke volume at 30 days (TriAlign) Improvement in NYHA functional class at 30 days (TriAlign) and 12 months (Cardioband) ↑ 6MWD at 30 days (TriAlign) and 6 months (Cardioband)	[16,17]
Edge-to-Edge Repair					
	TriClip (Abbott, USA) PASCAL (Edwards Lifesciences, USA)	Femoral vein		↓ Tricuspid regurgitation at 12 months (TriClip) Improvement in NYHA functional class at 12 months (TriClip) ↑ 6MWD at 12 months (TriClip) ↑ KCCQ at 12 months (TriClip)	[18,19]
Tricuspid Valve Replacement					
Caval Techniques	TricValve system (P&F, Germany)	Femoral vein	≥3+ tricuspid regurgitation NYHA functional class III–IV	Improvement in NYHA functional class at 6 months (TricValve) ↑ KCCQ at 6 months (TricValve) ↓ Hepatic vein backflow at 6 months (TricValve)	[20]
Ventricular Restoration Devices					
	Parachute device (CardioKinetix, USA) AccuCinch (Ancora Heart, USA) Revivent TC system (BioVentrix, USA)	Femoral artery (Parachute device) Femoral artery (AccuCinch) Median sternotomy or hybrid mini thoracotomy + right internal jugular vein (Revivent)	NYHA functional class II–IV symptoms (PARACHUTE) LVEF 15–40% (PARACHUTE) Dilated, akinetic, or dyskinetic anterior apex (PARACHUTE) NYHA functional class II–IV symptoms (Revivent) LV dilation (Revivent) Transmural scar in anteroseptal, anterolateral, or apical segments (Revivent)	↓ End diastolic volume index at 36 months (PARACHUTE) ↑ LVEF at 12 months (Revivent) ↓ LVESVi at 12 months (Revivent) ↓ LVEDVi at 12 months (Revivent) Improvement in NYHA functional class at 12 months (Revivent) ↑ 6MWD at 12 months (Revivent)	[21,22]
Interatrial shunt devices					
	Interatrial shunt device (IASD) (Corvia Medical, USA) V-wave shunt device (V-wave Ltd., Israel)	Femoral vein	NYHA functional class III or IVa (IASD) LVEF ≥ 40% (IASD) Exercise PCWP ≥ 25 mmHg (IASD) PCWP—RAP ≥ 5 mmHg (IASD)	↓ Annual rate of HF hospitalization at 12 months (REDUCE LAP I) ↑ Right ventricular dimensions at 6 months (REDUCE LAP I) No reduction in CV death or non-fatal ischemic stroke at 12 months, HF events at 24 months, or KCCQ at 12 months (REDUCE LAP II)	[23,24]

## Data Availability

Not applicable.
